# Nuclear-targeted siRNA delivery for long-term gene silencing†
†Electronic supplementary information (ESI) available. See DOI: 10.1039/c6sc04293g



**DOI:** 10.1039/c6sc04293g

**Published:** 2017-01-19

**Authors:** Na Li, Huijun Yang, Zhengze Yu, Yanli Li, Wei Pan, Hongyu Wang, Bo Tang

**Affiliations:** a College of Chemistry , Chemical Engineering and Materials Science , Collaborative Innovation Center of Functionalized Probes for Chemical Imaging in Universities of Shandong , Key Laboratory of Molecular and Nano Probes , Ministry of Education , Institute of Molecular and Nano Science , Shandong Normal University , Jinan 250014 , P. R. China . Email: tangb@sdnu.edu.cn

## Abstract

Developing effective nonviral siRNA delivery systems for long-term gene silencing remains a great challenge. Here we present a nuclear-targeted siRNA delivery system that can induce long-term gene silencing in cancer cells. The nanocarrier consists of gold nanoparticles modified with a dense shell of synthetic siRNAs and nuclear localization signal (NLS) peptides. The NLS peptide could translocate the nanocarrier into the nucleus and the siRNA was designed to target the promoter of thymidine kinase 1 and trigger the RNA-directed DNA methylation, thereby enabling the nuclear-targeted gene silencing. Compared with traditional gene silencing in cytoplasm, long-lasting gene knockdown could be achieved for the nuclear-targeted nanocarrier, which lasts for more than 30 days. The long-term gene silencing induced by nuclear-targeted siRNA delivery could effectively inhibit the proliferation of cancer cells and prevent the formation of a tumor in a mouse model.

## Abstract 2

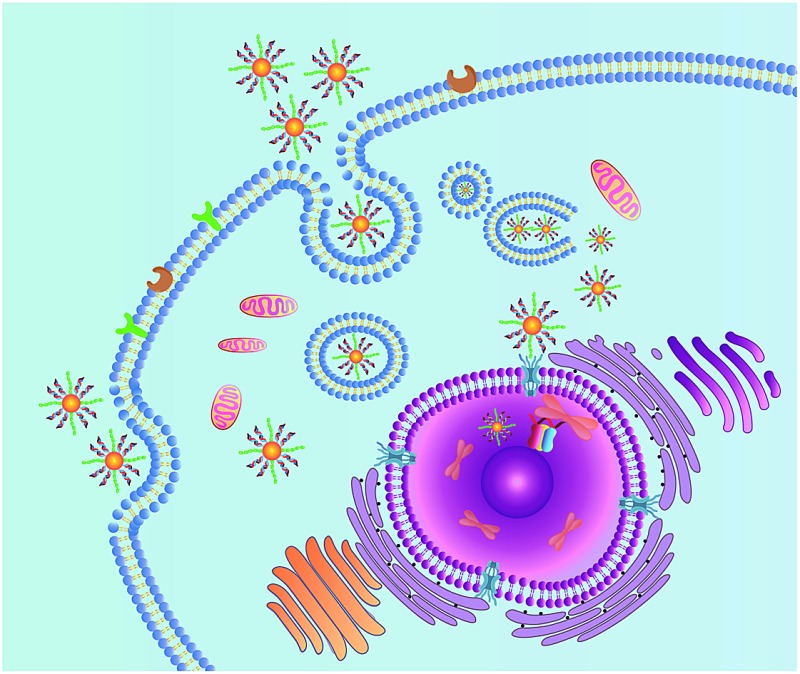
A nuclear-targeted siRNA delivery system was developed for long-term gene silencing in cancer cells. The nanocarrier consists of gold nanoparticles, synthetic siRNAs and nuclear localization signal peptides.

## Introduction

RNA interference (RNAi) is a very powerful tool, which can regulate the expression of specific target genes.^1^ RNAi pathways have the potential to silence disease-causing genes as a therapeutic approach.^2^ Synthetic small interfering RNA (siRNA) is a class of double-stranded RNA molecules, which can direct the post-transcriptional degradation of complementary mRNA or transcriptional gene silencing by methylation of homologous DNA sequences.^3–7^ In recent years, siRNA has emerged as a promising therapeutic modality to knock down disease-related genes. Many efforts have been made to explore siRNA delivery systems by using viruses or nonviral vectors, such as liposomes, polycationic polymers,^8^ conjugates,^9^ and nanoparticles.^10–12^ Viral vectors have shown high efficiency in siRNA delivery *in vivo*, however, delivery using viruses may result in harmful immune-mediated responses, unwanted vector uptake, and other toxic side effects.^13^ Although the non-viral based gene delivery systems exhibit several advantages over viral systems, the therapeutic effect is often limited by their poor ability for long-lasting and complete knockdown.^8,10,14^ To acquire a persistent silencing effect *in vivo*, repeated administration of the agents is usually required.^9^ Such repeated injections can lead to a substantial impediment for patient treatment, as verified by reduced enrollment in clinical trials and decreasing patient compliance. The development of effective siRNA delivery systems for complete and prolonged gene silencing with a single treatment is highly desirable to improve the knockdown effect and decrease the administration frequency.

The cell nucleus is the control centre of the cell, which maintains genetic integrity and controls cellular activities by regulating gene expression.^15–20^ Considering that the nucleus is the most important organelle and the destination of genetic information, transporting the therapeutic agents to the nucleus can enormously improve the efficiency of gene therapy. A nuclear localization signal (NLS) peptide^21–26^ has been employed as an effective targeted moiety for nuclear localization, which is capable of delivering the nanocarrier into the cell nucleus. Therefore, the NLS peptide could transport the siRNA delivery system into the nucleus and ensure that the gene silencing directly operates in the correct place.

**Scheme 1:**
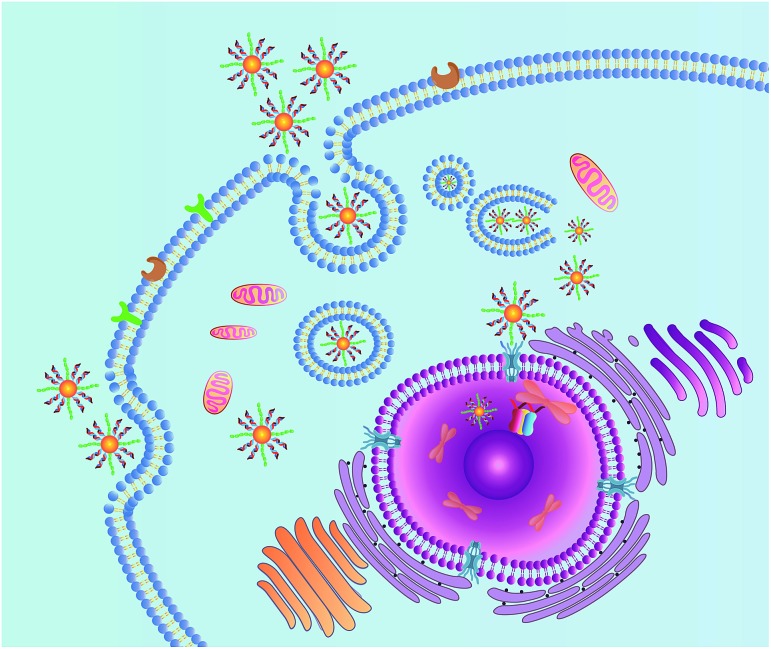
Schematic illustration of the nanocarrier for nuclear-targeted siRNA delivery.

Herein, we demonstrate a novel and effective strategy to construct a nuclear-targeted nanocarrier for siRNA delivery in cancer cells. The nanocarrier consists of gold nanoparticles (AuNPs) assembled by gold–thiol bonds with a dense shell of synthetic siRNAs. The nucleic acid conjugated AuNPs possess good dispersibility in aqueous systems, decreased immunogenicity and good biocompatibility.^27^ The nuclear targeting can then be achieved by functionalization of the NLS peptide on the surface of the AuNPs. When the nanocarrier enters the cell *via* receptor-mediated endocytosis, the NLS peptide can interact with the nuclear pore complex and translocate the nanocarrier into the cell nucleus. The nucleus is a satisfactory place for complete gene silencing, because the cellular genetic information and transcription machinery reside there.^15^ Once the siRNAs loaded by the nanocarrier are introduced into the nucleus, the siRNAs are capable of targeting the promoter of Thymidine Kinase 1 (TK1), which is closely associated with cell division and is considered to be an important marker for tumor growth.^28,29^ RNA-directed DNA methylation can be initiated, which could induce long-term transcriptional silencing of homologous promoters.^5^ The details of this approach are shown in Scheme 1.

## Results and discussion

To achieve nuclear targeting, 13 nm AuNPs were utilized for the nanocarrier because of their suitable size. The siRNA (TK1Pro siRNA) was designed to target the promoter of TK1. The nanocarrier was then synthesized by mixing the AuNPs with the siRNAs and the NLS peptides by a two step method. The detailed information of the siRNA and peptide is shown in Table S1.† The TEM images of the AuNPs and the nanocarrier with the NLS peptide (NWN) are shown in Fig. S1 in ESI.† The UV-vis absorption spectra (Fig. S1d, ESI†) confirmed the successful assembly of the nanocarrier due to the red shift from 519 nm for the AuNPs to 523 nm for the nanocarrier.^30,31^ The electrophoresis result also indicated that the siRNAs were modified on the surface of the AuNPs. The number of siRNAs per AuNP was calculated to be 22 ± 1 by a nanodrop experiment.^32^

The stability of the designed gene delivery system in cells and serum was evaluated first. Three groups of NWN were incubated in PBS, cell and serum for 2 h, respectively. NWN in PBS, cell lysate and serum was then separated by centrifugation and treated with dithiothreitol (DTT) to separate the siRNA from the gold nanoparticles. The RNA levels in the supernate were measured using a nanodrop-based method to investigate its stability. As shown in Fig. S2,† the RNA levels in cell lysate and serum were almost the same as those in PBS, which indicated the good stability of NWN under physical conditions.

**Fig. 1:**
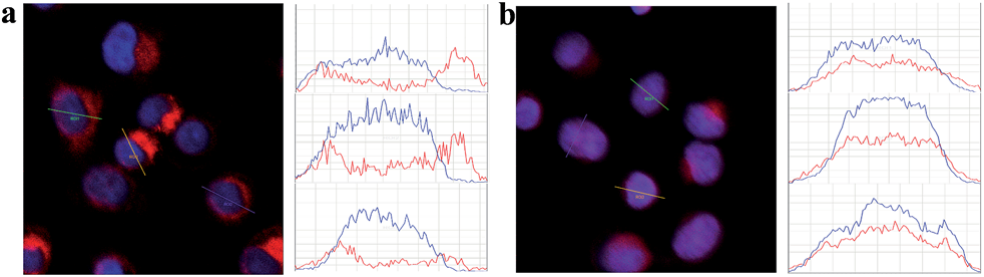
CLSM images of MCF-7 cells incubated with 3 nM NWON-Cy5 (a) and with 3 nM NWN-Cy5 (b). Right column shows the fluorescence intensity profiles across the lines in (a) and (b).

To estimate the potential for nuclear-targeted gene suppression by the nanocarrier through non-invasive delivery, we then studied its uptake in the nucleus using confocal laser scanning microscopy (CLSM). The nanocarrier without NLS peptides (NWON) was employed for comparison. To trace the nanocarrier, Cy5 was modified at the end of the siRNA. Human breast cancer cell line (MCF-7) was chosen as an example and was incubated with the 3 nM nanocarrier with and without NLS. Nuclear staining was then employed to evaluate the nuclear targeting ability. As shown in Fig. 1, NWON was distributed in the cell cytoplasm randomly, while most of NWN was located in the nucleus, suggesting that NWN exhibits excellent ability for nuclear targeting. The line scanning profiles of the fluorescence intensity of the different cells further qualitatively confirmed the above observations.

**Fig. 2:**
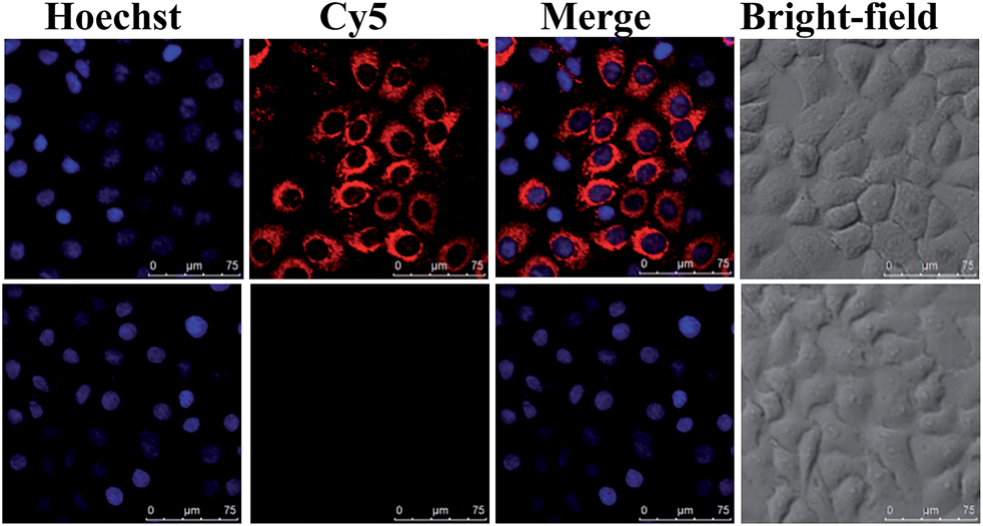
Confocal imaging of TK1 mRNA expression. MCF-7 cells were treated with (bottom panels) and without (top panels) NWN (5 nM, 48 h) before they were incubated with the nanoprobes (1 nM) for the detection of TK1 mRNA.

We next evaluated the ability of NWN to silence gene expression in various cancer cells, including MCF-7, cervical cancer cell line (HeLa), and human hepatocellular liver carcinoma cell line (HepG2). Real-time PCR (RT-PCR) results showed that the expression of TK1 mRNA decreased to about 10% for all three kinds of cells when they were incubated with NWN for two days, demonstrating that NWN possessed tremendous RNAi efficiency and achieved robust gene silencing (Fig. S3, ESI†). When the three kinds of cells were treated with NWON or SNWN, the expressions of TK1 mRNA all showed a slight decrease to about 90%, which were of comparable magnitude to the controls. The results indicated that the NLS peptides and siRNA played decisive roles in the nuclear-targeted gene silencing. To further confirm the silencing of TK1 mRNA by NWN, a nanoprobe based on the AuNPs and molecular beacons (MBs) was synthesized according to our previous report.^33^ As shown in Fig. 2, a strong red fluorescence signal for TK1 mRNA was observed for the untreated MCF-7 cells. A very faint red fluorescence signal was obtained for the NWN-treated cells, suggesting that the TK1 mRNA was indeed silenced by NWN, which was consistent with the RT-PCR results.

Subsequently, PCR experiments were conducted to study the specificity of the designed NWN and investigate whether it would cause unexpected off-target effects. As shown in Fig. S4,† the expression of TK1 mRNA decreased to approximately 10% when the MCF-7 cells were treated with NWN compared to that of untreated cells. However, the expression of chloramphenicol acetyltransferase (CAT) mRNA remained at its initial level and no obvious decrease appeared. The results revealed that NWN could specifically downregulate TK1 expression and had no influence on other TK1 promoter-related genes.

**Fig. 3:**
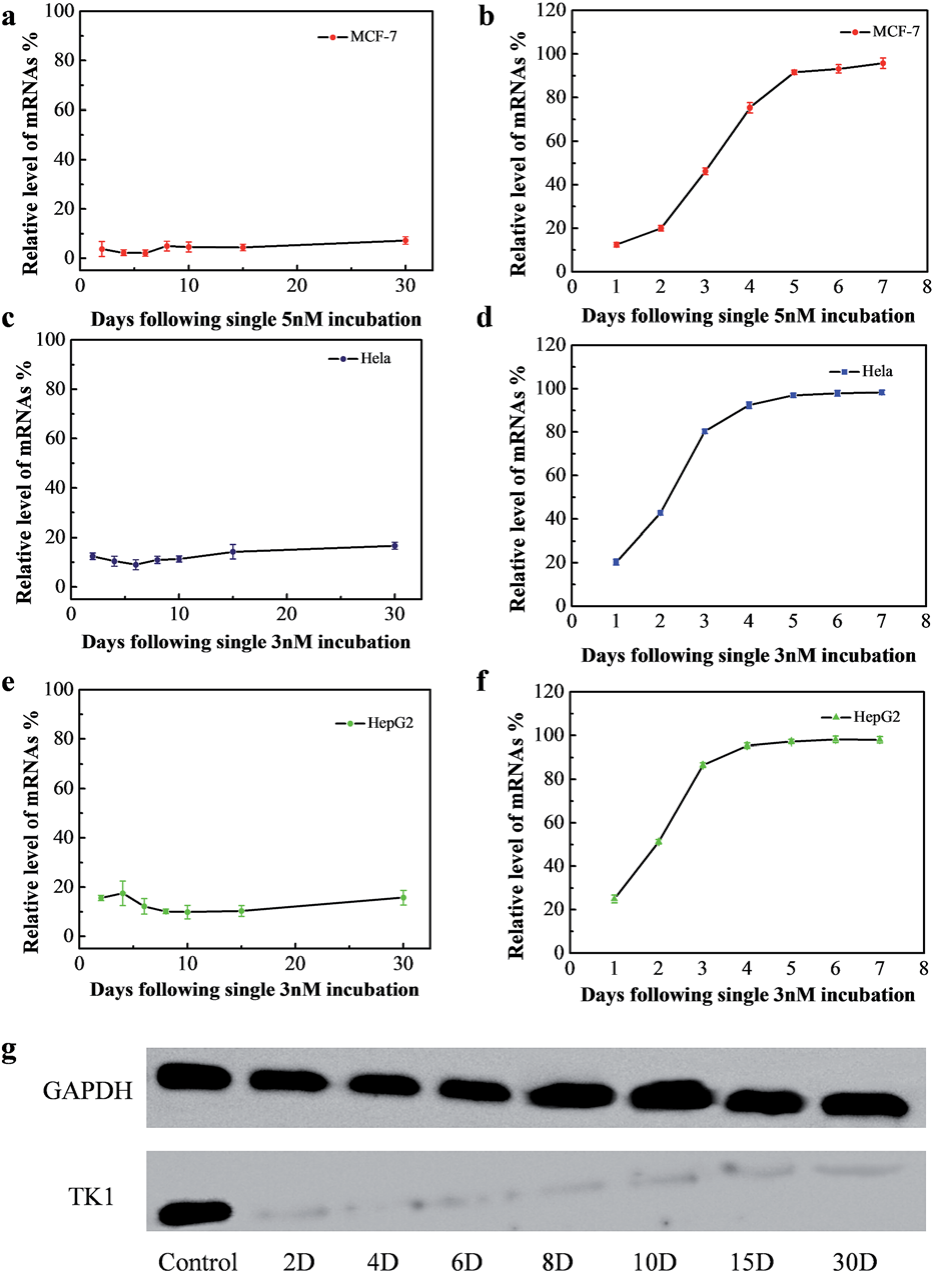
(a), (c) and (e) Gene silencing effect of NWN for different days. MCF-7, HeLa and HepG2 cells were incubated with 5 nM or 3 nM NWN for two days. (b), (d) and (f) Gene silencing effect of NTM for different days. MCF-7, HeLa and HepG2 cells were incubated with 5 nM or 3 nM NTM for two days. (g) Western blotting was carried out during a one month period after NWN treatment. MCF-7 cells were incubated with 5 nM NWN for two days.

The long-term gene silencing effect of NWN was then evaluated. Firstly, the cells were incubated with NWN for two days. The expression of TK1 mRNA of the treated cells was evaluated on different days (from 2 days to 30 days) by RT-PCR. TK1 mRNA expression in MCF-7 initially decreased to 4% at 2 days and remained suppressed between 2 and 7% for 30 days without further addition of NWN (Fig. 3a). Western blot results further confirmed that the TK1 protein expression was almost completely suppressed for all 30 days (Fig. 3g). Next, HeLa and HepG2 cells were studied to demonstrate the universal application of the nanocarrier during the gene silencing process. Notably, the TK1 mRNA expression in the two cells was suppressed to 10–17% at 30 days (Fig. 3c and e), suggesting that NWN is capable of long-term gene silencing. To evidence the pivotal role of nuclear targeting in long-term gene silencing, the nanocarrier with the siRNA (CTK1 siRNA) targeted to TK1 mRNA in the cytoplasm (NTM) was also prepared as a comparison. NTM was also incubated with MCF-7 cells under the same conditions and the RT-PCR results showed that the expression of TK1 mRNA in MCF-7 cells decreased to 12% at 1 day, while it changed to 54% at 3 days and the suppression was completely lost at 5 days (Fig. 3b). The NTM was then incubated with HeLa and HepG2 cells, in which the suppression was also completely lost at 4–5 days (Fig. 3d and f). These results were similar to those of the previous report,^8,10^ suggesting that traditional gene silencing in the cytoplasm is not sustained over a long time. Taken together, our studies demonstrate that nuclear-targeted gene silencing has better durability and stability for gene silencing compared with the gene silencing in the cytoplasm.

**Fig. 4:**
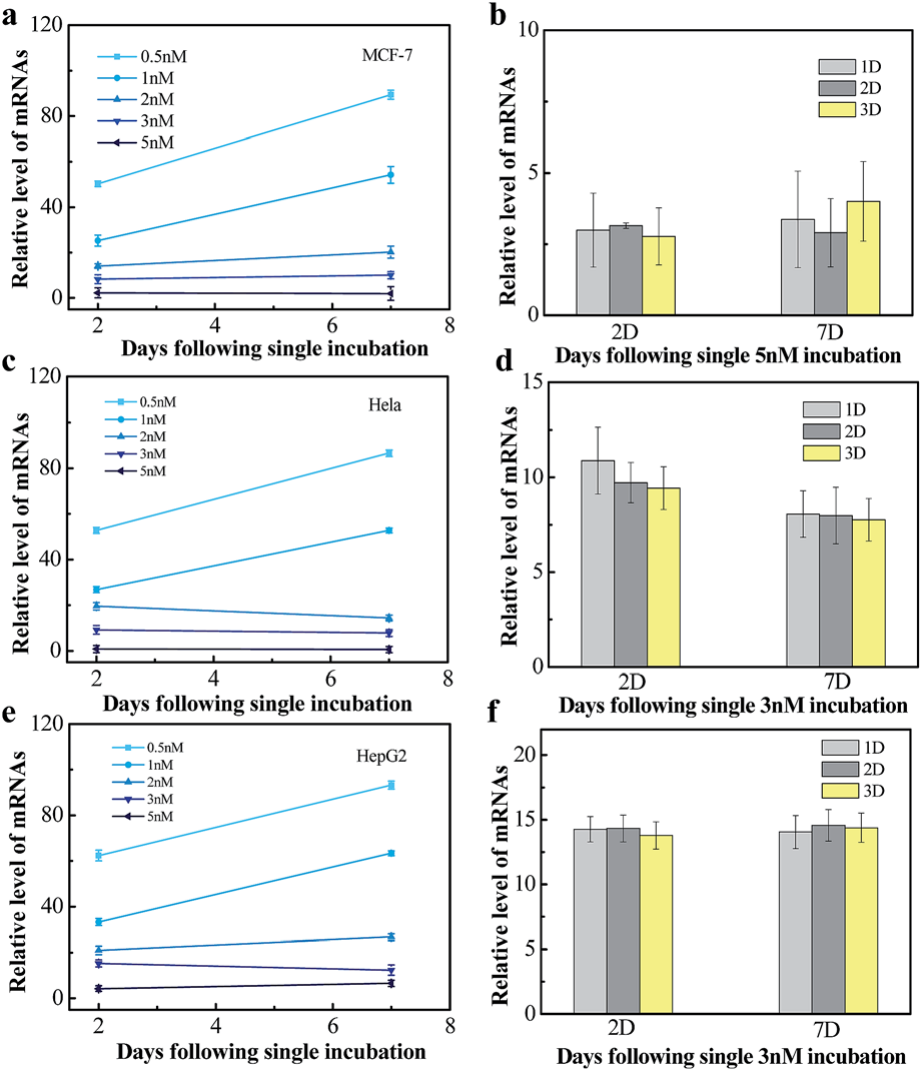
(a), (c) and (e) Dose dependence of 7 day gene silencing in MCF-7, HeLa and HepG2 cells. All of the cells were incubated with 0.5, 1, 2, 3 and 5 nM NWN. (b), (d) and (f) TK1 mRNA expression on day 2 and day 7 in MCF-7, HeLa and HepG2 cells. The cells were incubated with 5 nM or 3 nM NWN.

We next tested the influence of incubation dose of NWN and time on the long-term gene silencing effect. MCF-7, HeLa and HepG2 cells were incubated with different concentrations of NWN (0.5, 1, 2, 3, and 5 nM) for two days. RT-PCR was carried out at 2 days and 7 days after incubation. As shown in Fig. 4a, when treated with 0.5 nM NWN, the expression of TK1 mRNA in the MCF-7 cells was initially decreased to 50% but the suppression was abolished at 7 days. The initial suppression effect was enhanced with the increase of NWN concentration. When the NWN concentration was above 2 nM, the expression of TK1 mRNA was suppressed to less than 20% and long-term gene silencing was triggered. The gene silencing could be maintained for more than 30 days and the expression of TK1 mRNA in this period kept constant at a low level as mentioned above (Fig. 4a). Similar phenomena were also observed in HeLa cells and HepG2 cells (Fig. 4c and e). These results suggested that the initial suppression decreased to about 20% and durable silencing could be triggered when the concentration of NWN reached a threshold (2 nM for this study). Compared with incubation dose, incubation time barely influenced the silencing effect (Fig. 4b, d and f).

To determine the internalization pathway for NWN into living cells, various endocytosis inhibitors were employed.^8,34^ The results showed that NWN might be mainly internalized *via* caveolae-mediated and clathrin-mediated endocytic pathways (Fig. S5 and 6 ESI†).

**Fig. 5:**
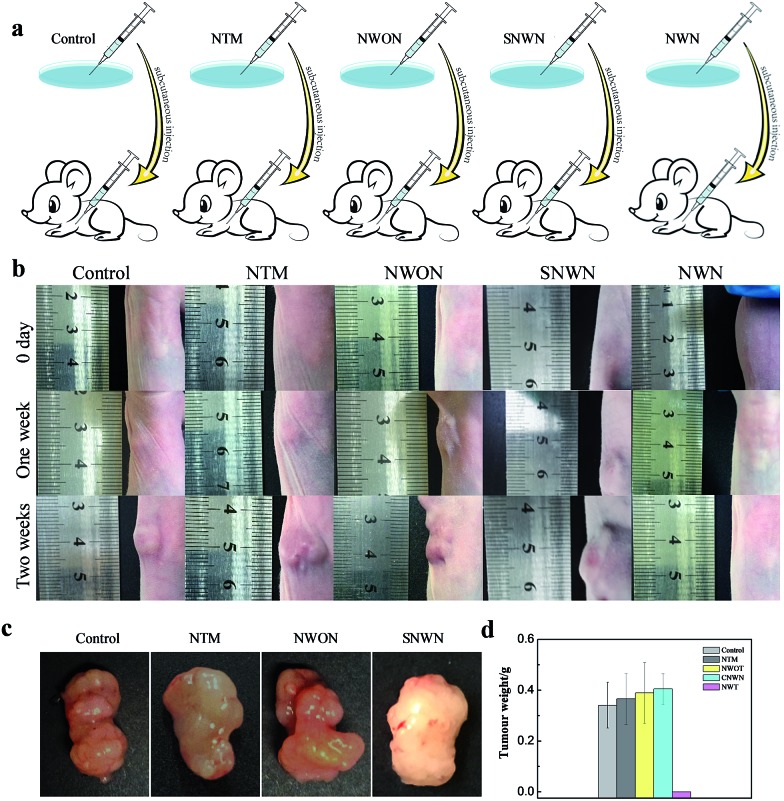
(a) Schematic illustration of *in vivo* experiment. Nude mice were subcutaneously injected with MCF-7 cells treated with NWON, NTM or NWN. Tumors were harvested two weeks later. (b) The mice were subcutaneously injected with MCF-7 cells with different treatment and the tumor size was monitored during the 14 day period. (c) Representative tumors separated from nude mice subcutaneously injected with MCF-7 cells with different treatment. (d) Average weight of the tumors.

We then sought to investigate the effect of long-term silencing of TK1 on the formation of a tumor in a mouse model. TK1 is associated with cell division and is proposed to be a marker of tumor growth.^35–38^ Although the previous report indicated that the silencing of TK1 in the cytoplasm would not affect the proliferation of cells,^29^ we found that durable gene silencing of TK1 in the nucleus was critical for the proliferation of MCF-7 cells. The proliferation speed of MCF-7 cells slowed down obviously after the treatment with the nanocarrier (Fig. S7, ESI†). However, the proliferation speed changed little for the normal breast cells (MCF-10A) and hepatocyte line (HL-7702) (Fig. S8 and 9, ESI†), because TK1 is not over-expressed in these normal cells. Therefore, we speculated that the long-term gene silencing might prevent the tumor from forming. Next, three groups of nude mice were subcutaneously injected with the NWN treated cells, the NWON treated cells and untreated cells, respectively. Two weeks later, the mice were sacrificed and the tumors were harvested to record the weight. The tumors were formed and developed rapidly in the NWON treated group and untreated group. But tumors were not found in the NWN treated group as expected (Fig. 5). In order to confirm the crucial role of durable silencing of TK1 in preventing tumor formation, NTM and NLS conjugated AuNPs using scrambled siRNA (SNWN) were also employed. The tumors also formed after two weeks for the two groups. The results were consistent with those of a previous report,^29^ indicating that TK1 deletion in the cytoplasm would not affect the proliferation of cells and the formation of a tumor. It was concluded that nuclear-targeted siRNA delivery was the key factor for long-term gene silencing and affected the ability of tumor cells, which could prevent the formation of a tumor in a mouse model.

The tumor growth inhibition efficacy of the designed NWN *in vivo* was also evaluated. Mouse models with xenograft tumors were obtained by treating with untreated MCF-7 tumor cells. NWN was injected intratumorally when the volume of the solid tumor reached about 80–100 mm^3^. The tumor sizes were monitored during the treatments. As shown in the photographs, the tumors treated with NWN were suppressed compared to the control (Fig. S10a†). The tumor weights of the control group showed about a 2.5-fold increase compared to those of the group treated with the designed NWN at two weeks post-treatment (Fig. S10b and c†). These results indicated that nuclear-targeted siRNA delivery could inhibit the tumor growth efficiently.

## Conclusions

In summary, we have developed a nuclear-targeted siRNA delivery platform based on gold nanoparticles, which can be used for long-term gene silencing in cancer cells. Compared with the nanocarrier without nuclear targeting, the expression of TK1 mRNA was greatly reduced for the nuclear-targeted nanocarrier. Moreover, the nuclear-targeted siRNA delivery could efficiently suppress the TK1 protein and TK1 mRNA expression for more than 30 days. The comparison test of gene silencing in the cytoplasm indicated that nuclear targeting plays a pivotal role in long-term gene silencing. Significantly, the proliferation of cells and the formation of a tumor *in vivo* were enormously inhibited after treatment with the nuclear-targeted nanocarrier. We expect that the current strategy will offer new opportunities for long-term gene silencing and cancer therapy.

## Experimental section

### Synthesis of gold nanoparticles

The gold nanoparticles (AuNPs) of 13 nm were prepared using the method reported before.^38^ All glassware was cleaned in aqua regia (HCl/HNO_3_, 3 : 1) and rinsed with H_2_O. 100 mL HAuCl_4_ (0.01%) was heated to boiling with vigorous stirring, then 2.0 mL of trisodium citrate (1%) was added under stirring. The color of the solution turned from pale yellow to colorless and finally to burgundy. Boiling was continued for an additional 10 min. The colloid was stirred until the solution reached room temperature. After that it was filtered through a 0.45 μm Millipore membrane filter. Transmission electron microscopy (TEM) images indicated that the particle sizes are 13 ± 2 nm (100 particles sampled). The prepared AuNPs were stored at 4 °C.

### Preparation of the nanocarrier

A AuNP colloid solution (3 nM) was treated with an RNA secure reagent. Duplex siRNA (150 nM) was then incubated with the RNA secure treated AuNP colloid and was continued to be shaken. After 6 h, an RNA secure treated SDS solution (0.1%) was added to the mixture to reach a final concentration of 0.01% and shaken overnight. The salt concentration of the mixture was then increased from 0.1 to 0.2 M during the aging process. Sonication was used to increase the coverage of oligoribonucleotides on the surface of the AuNPs. After 48 h aging, the AuNPs were purified by centrifugation at 13 000 rpm for 30 min at 4 °C, and resuspended in RNase-free PBS (10 mM). The process was repeated three times. 1.5 μM NLS peptides were then added to the solution, and the mixture was shaken for 48 h. After functionalization, the nanocarrier was purified by centrifugation at 13 000 rpm for 30 min at 4 °C, and resuspended in RNAse-free PBS (10 mM). The process was repeated three times.

### Preparation of TK1 mRNA nanoprobe

The molecular beacon (150 nM) targeting TK1 mRNA was added to the AuNP colloid (3 nM) and was kept stirring. After 6 h, SDS solution (0.1%) was added to the mixture to reach a final concentration of 0.01% and stirred overnight. The salt concentration of the mixture was then increased from 0.1 to 0.2 M during the aging process. Sonication was used to increase the coverage of oligoribonucleotides on the surface of the AuNPs. After 48 h aging, the nanoprobe was purified by centrifugation at 13 000 rpm for 30 min at 4 °C, and resuspended in PBS (10 mM).

### Nanodrop experiment

ME (20 mM) was added to 1 nM nanocarrier, and the mixture was stirred overnight. After this, centrifugation was carried out and a supernate was obtained. Nanodrop 2000 was used to obtain the concentration of the RNA in the supernate,^32^ then the number of the siRNA on each nanocarrier was determined by: RNA concentration/AuNP concentration.

### Stability of the nanocarrier

Three groups of NWN were first incubated in PBS, cell and serum for 2 h, respectively. NWN in PBS, cell lysate and serum was then separated by centrifugation and treated with dithiothreitol (DTT, 20 mM) for 4 h to separate the siRNA from the gold nanoparticles. The RNA levels in the supernate were measured using a nanodrop-based method.

### Nuclear targeting experiment

A Cy5 fluorochrome was tagged on the 5′ end of the TK1Pro siRNA. The MCF-7 cells were incubated with the nanocarrier with or without NLS for 6 h at 37 °C. Hoechst 33342 was added into the medium before imaging. The cells were then washed with PBS three times, and the images were acquired using CLSM with an objective lens (×20).

### Gene silencing effect of the nanocarrier with or without NLS

All of the cells were incubated with 3 nM nanocarrier with or without NLS. After two days of incubation, the nanocarrier was washed away and fresh medium was added. RT-PCR was performed two days later. MCF-7 cells treated with or without the nanocarrier were incubated with the nanoprobe targeting TK1 mRNA for 4 h. CLSM was then used to monitor the expression of TK1 mRNA under 633 nm excitation.

### Intracellular gene silencing of the nanocarrier

RT-PCR was used to evaluate the gene silencing effect. All of the cells were divided into two groups, one had no treatment and the other was incubated with nanocarrier. MCF-7 cells were incubated with 5 nM nanocarrier, and HepG2 and HeLa cells were incubated with 3 nM nanocarrier. After two days of incubation, the nanocarrier was washed away and fresh medium was added. RT-PCR was performed at 2, 4, 6, 8, 10, 15 and 30 days.

### Western blot experiment

MCF-7 cells were used to perform the western blot experiment. The cells were incubated with 5 nM nanocarrier for two days. After two days of incubation they were replaced with fresh medium. 10^6^ cells were collected and resuspended in 800 μL of RIPA Lysis containing protease and phosphatase inhibitor (Thermo Scientific). The total protein concentration was determined by the Pierce BCA protein assay kit (Thermo Scientific). Equal amounts of protein samples were fractionated by a 4–20% pre-cast gradient gel, transferred to nitrocellulose membranes and then it was blocked with Odyssey blocking buffer (LI-COR Biosciences). Thymidine Kinase 1 (TK1) and GAPDH were probed by primary rabbit antibody (Abcam) against TK1 and GAPDH, respectively. After that, the membranes were incubated with horseradish peroxidase-conjugated goat anti-rabbit immunoglobulin G (IgG) (Santa Cruz Biotechnology, INC., Santa Cruz, California, USA), then processed using the enhanced chemiluminescence detection system, Luminescent Image Analyzer LAS-4000mini (Fujifilm, Tokyo, Japan).

### Gene silencing effect of nanocarrier targeted mRNA

The nanocarrier was prepared with the duplex siRNA target mRNA of TK1, and the NLS peptides were deleted from the nanocarrier. All of the cells were divided into two groups, one had no treatment and the other was incubated with the nanocarrier. MCF-7 cells were incubated with 5 nM nanocarrier and HepG2 and HeLa cells were incubated with 3 nM nanocarrier. After two days of incubation, the nanocarrier was washed away and fresh medium was added. RT-PCR was performed every day for one week.

### Evaluation of off-target effects

RT-PCR was used to evaluate off-target effects. MCF-7 cells were divided into two groups, one had no treatment and the other was incubated with 3 nM nanocarrier. After two days of incubation, the nanocarrier was washed away and fresh medium was added. The relative level of TK1 mRNA and chloramphenicol acetyltransferase (CAT) mRNA was measured after 2 days by RT-PCR. CAT, forward 5′-GCCTGACGATAGCAGCCTGGCGTACCGCTGGTGACTTCGCGTGTAGGCTGGAGCTGCTTC-3′. Reverse 5′-CCCGTTCAAAGCACTGCACCCCAGCCGAAAGCGGAGCCTGATGGGAATTAGCCATGGTCC-3′.

### Concentration dependence of incubation

All of the cells were divided into six groups and were incubated with 0, 0.5, 1, 2, 3, and 5 nM concentrations of the nanocarrier for 2 days, respectively. After incubation, the nanocarrier was washed away and fresh medium was added. RT-PCR was performed after 2 and 7 days.

### Time dependence of incubation

All of the cells were divided into four groups, one had no treatment and the other three groups were incubated with the nanocarrier for 1, 2 and 3 days respectively. MCF-7 cells were incubated with 5 nM nanocarrier and HepG2 and HeLa cells were incubated with 3 nM nanocarrier. After incubation, the nanocarrier was washed away and fresh medium was added. RT-PCR was performed after 2 and 7 days.

### Cellular uptake of the nanocarrier

Chlorpromazine was used to inhibit clathrin-mediated endocytosis (CME), filipin was used to inhibit cavolin-mediated endocytosis and dynasore was used to inhibit both pathways above. Ethylisopropylamiloride (EIPA) was used to inhibit macropinocytosis. All of the cells were divided into five groups, one group was set as a control group and the other groups were pre-incubated with endocytosis inhibitors: chlorpromazine (10 μM), filipin (5 μM), dynasore (100 μM) and EIPA (50 μM). The Cy5-tagged nanocarrier (3 nM) was then added into each group. After 4 h of incubation, confocal fluorescence imaging studies were carried out with an excitation wavelength of 633 nm.

### MTT assays

MCF-7 cells were cultured in 96-well microtiter plates and incubated at 37 °C in 5% CO_2_ for 24 h. The cells were then incubated with NWN (1, 3, and 5 nM) for two days. After incubation, the cells were washed with PBS buffer 3 times and incubated with fresh DMEM for different time lengths (1, 5, 7 and 14 days). The cells in the control group were without any treatment. 150 μL of MTT solution (0.5 mg mL^–1^) was then added to each well. After incubation for 4 h, the MTT solution was removed, and 150 μL of DMSO was added to each well under gentle stirring in the dark. MTT assays for MCF-10A cells and HL-7702 cells were carried out following the same procedure as above.

### *In vivo* experiments

(1) MCF-7 cells were divided into four groups, A, B, C and D. Group A without any treatment was set as the control. Group B was treated with 5 nM nanocarrier targeting TK1 mRNA. Groups C and D were treated with 5 nM nanocarrier with or without NLS for two days, then the nanocarrier was washed away and fresh medium was added. After two days, all of the cells were trypsinized and collected, then dispersed in 1× PBS buffer with a concentration of 5 × 10^6^ cells per mL. The nude mice were given a subcutaneous injection of 200 μL cell suspension from each group. Images were then taken each week and the tumors were harvested after two weeks. The morphology and weight of the tumors were recorded.

(2) Nude mice were inoculated with 1 × 10^6^ MCF-7 cells in PBS (150 μL), and divided into two treatment groups, A and B (*n* ≥ 5). When the tumor volume reached about 80–100 mm^3^ the tumor-bearing mice were weighed and randomly divided into two groups, A and B (*n* ≥ 5). Group A without any treatment was set as the control. Group B was treated with 3 nM nanocarrier (50 μL) targeting TK1 mRNA seven times at two day intervals.

## Conflict of interest

The authors declare no competing financial interests.
