# Supplementary material for: Nuclear-targeted siRNA delivery for long-term gene silencing
Source: Chem Sci. 2017 Jan 19;8(4):2816–22. doi: 10.1039/c6sc04293g (PMC5427680; doi:10.1039/c6sc04293g)
Supplement: Supplementary file 1 [file SC-008-C6SC04293G-s001.pdf]

Electronic Supplementary Material (ESI) for Chemical Science.
This journal is © The Royal Society of Chemistry 2017

Supplementary information

Nuclear-targeted siRNA delivery for long-term gene silencing

*Na Li, Huijun Yang, Zhengze Yu, Yanli Li, Wei Pan, Hongyu Wang and Bo Tang**

College of Chemistry, Chemical Engineering and Materials Science, Collaborative Innovation Center of Functionalized Probes for Chemical Imaging in Universities of Shandong, Key Laboratory of Molecular and Nano Probes, Ministry of Education, Shandong Provincial Key Laboratory of Clean Production of Fine Chemicals, Shandong Normal University, Jinan 250014, P. R. China

*e-mail: tangb@sdnu.edu.cn

Materials. DNA oligonucleotides were synthesized and purified by Sangon Biotechnology Co., Ltd (Shanghai, China). RNA oligonucleotides were synthesized and purified by TAKARA Biotechnology Co., Ltd. (Dalian, China). The sequence of the DNA and RNA oligonucleotides are listed in Table S1. Dynasore and Chlorpromazine were purchased from Sigma Chemical Company; Ethylisopropylamiloride (EIPA) was purchased from J&K Scientific Ltd., Filipin was purchased from Cayman Chemical Company; Trisodium citrate ($C_6H_5Na_3O_7{\cdot}2H_2O$), Hydrogen tetrachloroaurate (III) ($HAuCl_4{\cdot}4H_2O$, 99.99%) and mercaptoethanol (ME) were purchased from China National Pharmaceutical Group Corporation (Shanghai, China). Cell culture products, unless mentioned otherwise, were purchased from GIBCO. All the chemicals were of analytical grade and used without further purification. Sartorius ultrapure water (18.2 MΩ cm) was used throughout the experiments. The human breast cancer cell line MCF-7 was purchased from KeyGEN

biotechnology Company (Nanjing, China), the cervical cancer cell line HeLa was obtained from the Committee on Type Culture Collection of Chinese Academy of Sciences, and the Human hepatocellular liver carcinoma cell line HepG2 was obtained from the Committee on Type Culture Collection of the Chinese Academy of Sciences. The synthetic peptide was purchased from MoonBiochem Co., Ltd (Wuhan, china). RNAsecure was purchased from Beijing CoWin Biotech.

Instruments. Absorption spectra were measured on a pharmaspec UV-1700 UV-visible spectrophotometer (Shimadzu, Japan). Transmission electron microscopy (TEM) was carried out on a JEM-100CX II electron microscope. RT-PCR was carried out with LineGene 9620 sequence detection system (Bioer, Hangzhou, China). Confocal fluorescence imaging studies were performed with a TCS SP5 confocal laser scanning microscopy (Leica Co., Ltd. Germany) with an objective lens (×20). Nanodrop experiment was performed with a NanoDrop 2000 Spectrophotometer (Thermo Scientific).

Table S1

Oligonucleotide	Sequence
TK1Pro siRNA	5’-AAGCGGUCGGCGCGGGAACCAAAAAAA-$(CH_2)_3$-SH-3’
	3’-UUCGCCAGCCGCGCCCUUGGU-5’
Cy5-TK1Pro siRNA	5’-Cy5-AAGCGGUCGGCGCGGGAACCAAAAAAA-$(CH_2)_3$-SH-3’
	3’-UUCGCCAGCCGCGCCCUUGGU-5’
CTK1 siRNA	5’-AAGGCCUCAGGCCAGCCUGCCAAAAAA-$(CH_2)_3$-SH-3’
	3’-UUCCGGAGUCCGGUCGGACGG-5’
scramble siRNA	5’-AGGAGCUUGAAACCCGGUCGAAAAAAA-$(CH_2)_3$-SH-3’
	5’-UCGACCGGGUUUCAAGCUCCU-3’
TK1 MB	5’-Cy5-ACGACGCCAGGGAGAACAGAAACCGTCGTAAAAAA -$(CH_2)_3$-SH-3’
TK1 forward primer	5’-TATGCCAAAGACACTCGCTAC-3’
TK1 reverse primer	5’-GCAGAACTCCACGATGTCAG-3’
GAPDH forward primer	5’-GGGAAACTGTGGCGTGAT-3’
GAPDH reverse primer	5’-GAGTGGGTGTCGCTGTTGA-3’
CAT forward primer	5’-GCCTGACGATAGCAGCCTGGCGTACCGCTGGTGACTTCGCGTGTAGGCTGGAGCTGCTTC-3’
CAT reverse primer	5’-CCCGTTCAAAGCACTGCACCCCAGCCGAAAGCGGAGCCTGATGGGAATTAGCCATGGTCC-3’

Peptide	Sequence
NLS	CGGGGPKKKRKVGGGGPKKKRKVGGGGPKKKRKV

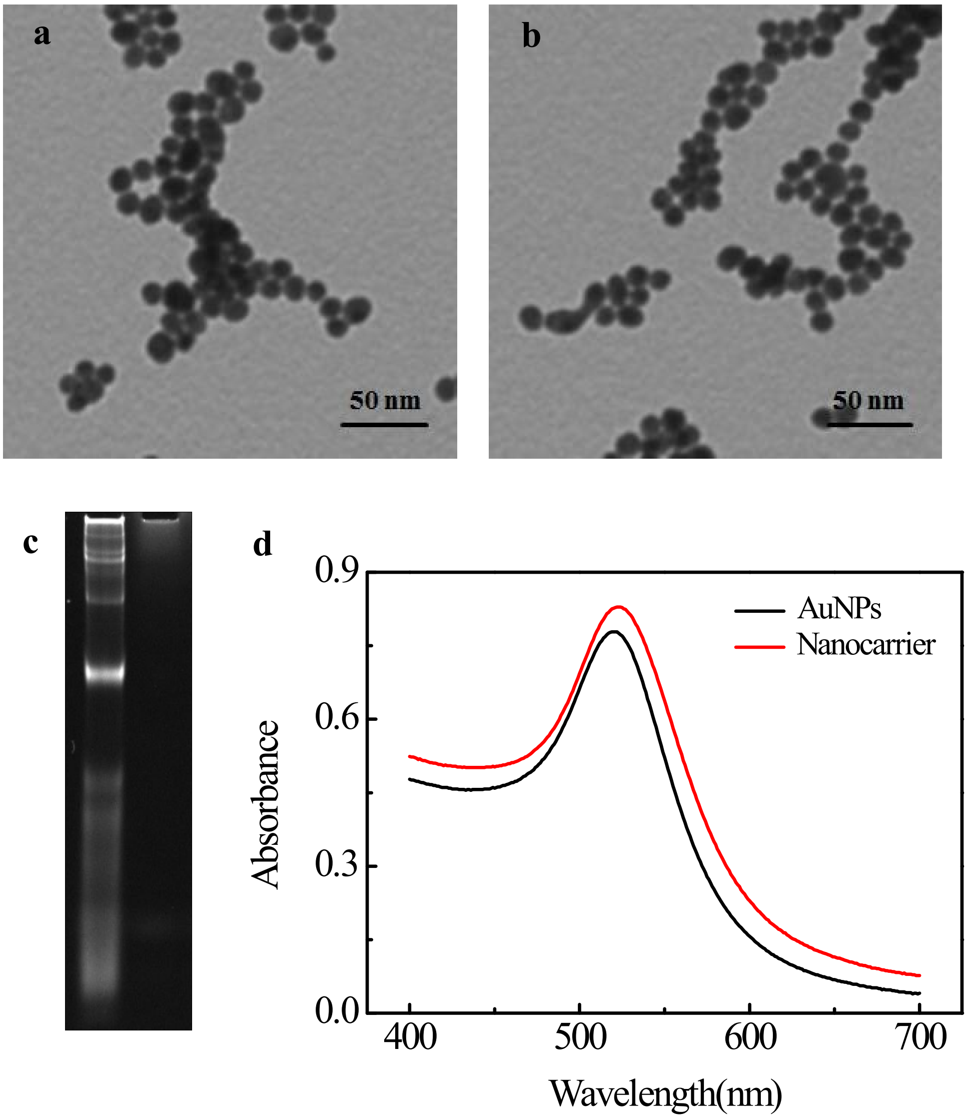

Figure S1. TEM images of (**a**) Au NPs and (**b**) nanocarrier. Scale bars are 50 nm. (**c**) Gel-electrophoresis of marker (left) and the nanocarrier (right). (**d**) UV-vis spectra for AuNPs and nanocarrier.

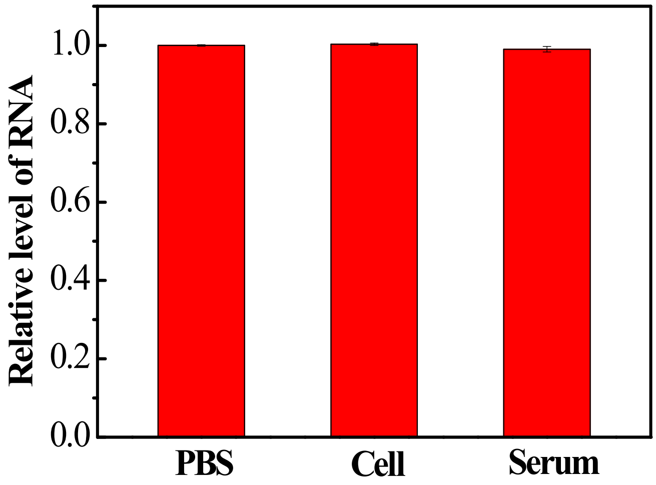

Figure S2. Relative level of RNA after NWN incubated in PBS, cell and serum.

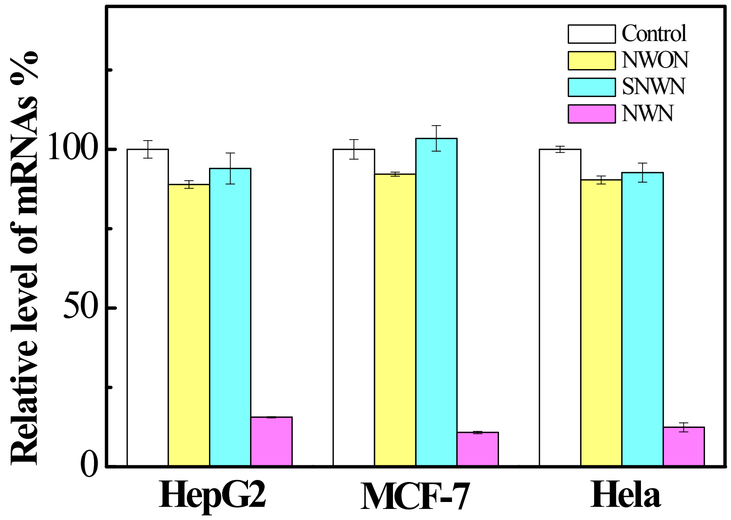

Figure S3. TK1 mRNA expression of different kinds of cells treated with NWN, SNWN or NWON for two days.

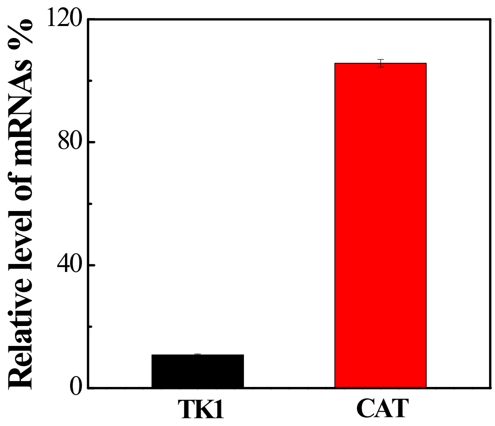

Figure S4. Relative level of TK1 and CAT mRNAs in MCF-7 cells treated with NWN compared to that in MCF-7 cells without treatments.

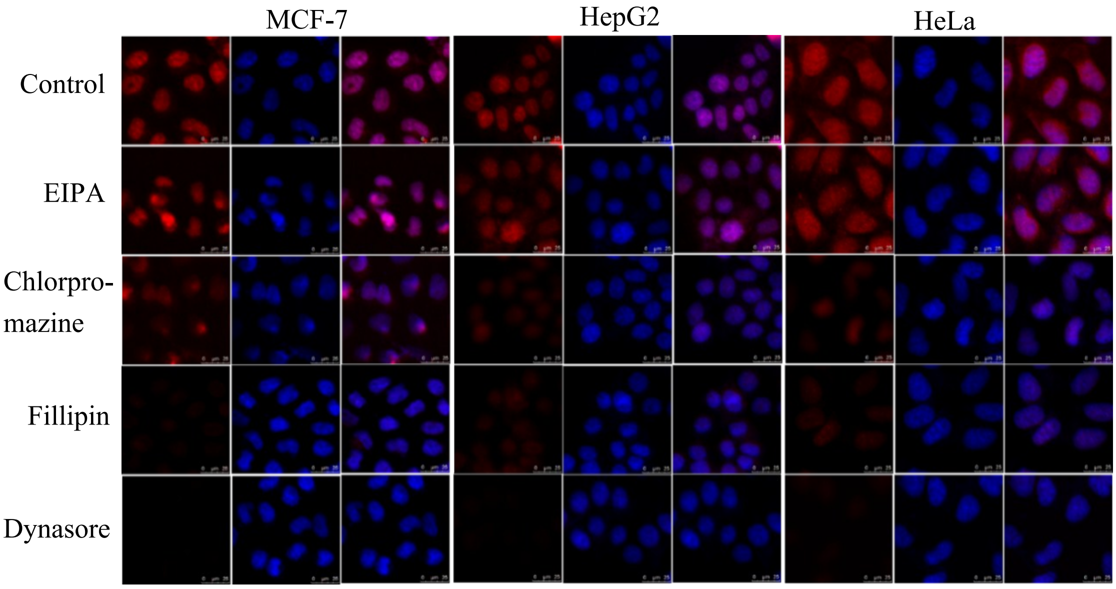

Figure S5. Confocal imaging of the different cells without treatment (control), treated with ethylisopropylamiloride (EIPA, 50 μM), chlorpromazine (10 μM), filipin (5 μM), and dynasore (100 μM) before incubated with NWN-Cy5.

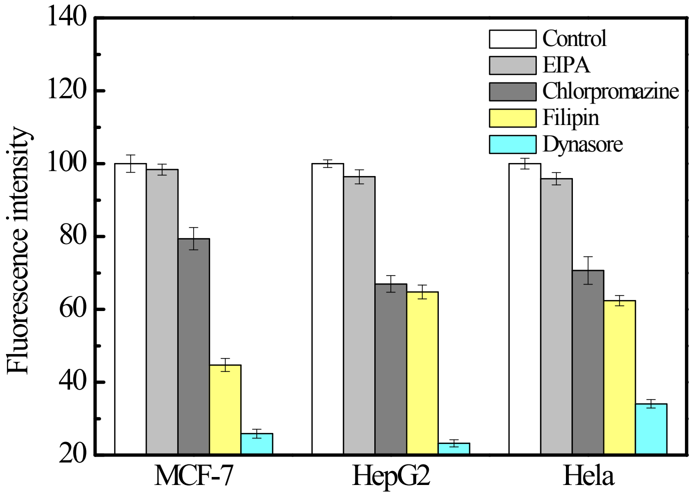

Figure S6. Cy5 fluorescence uptake in MCF-7, HepG2 and HeLa cells under NWN-Cy5 treatment in the presence of small molecules. Chlorpromazine: 10 μM, Filipin: 5 μM, Dynasore: 100 μM and EIPA: 50 μM.

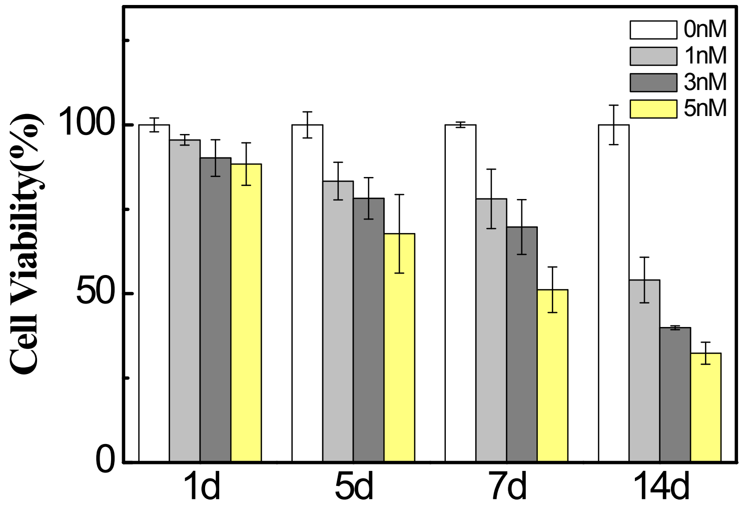

Figure S7. Cell viability for different days. The MCF-7 cells were incubated with 0, 1, 3 and 5 nM NWN.

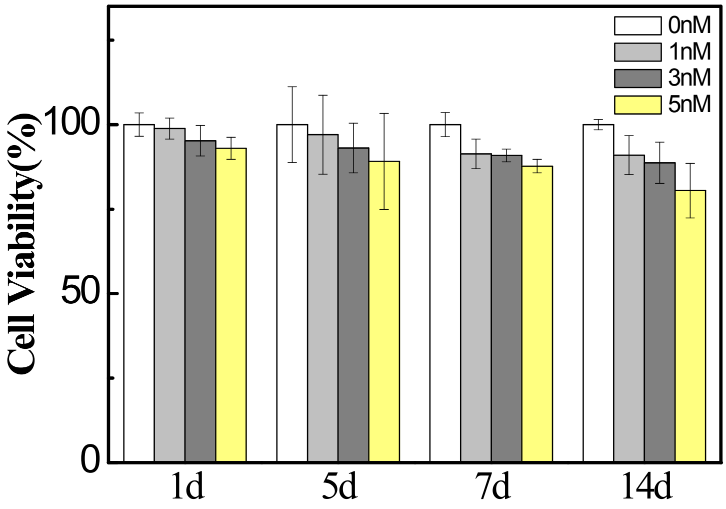

Figure S8. Cell viability for different days. The MCF-10A cells were incubated with 0, 1, 3 and 5 nM NWN.

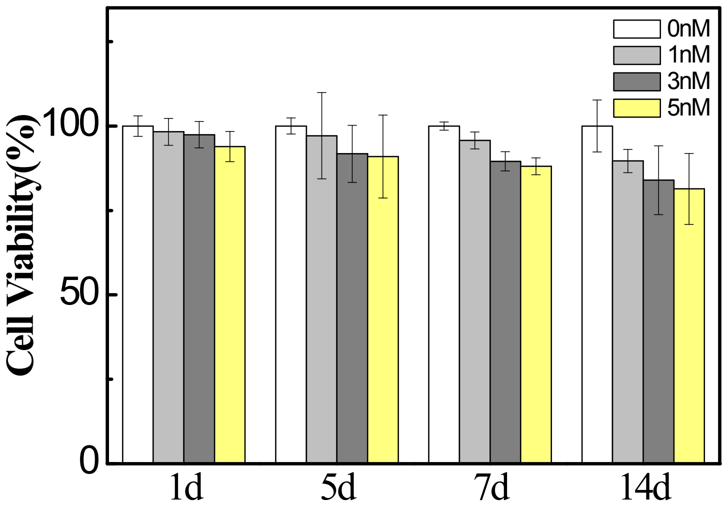

Figure S9. Cell viability for different days. The HL-7702 cells were incubated with 0, 1, 3 and 5 nM NWN.

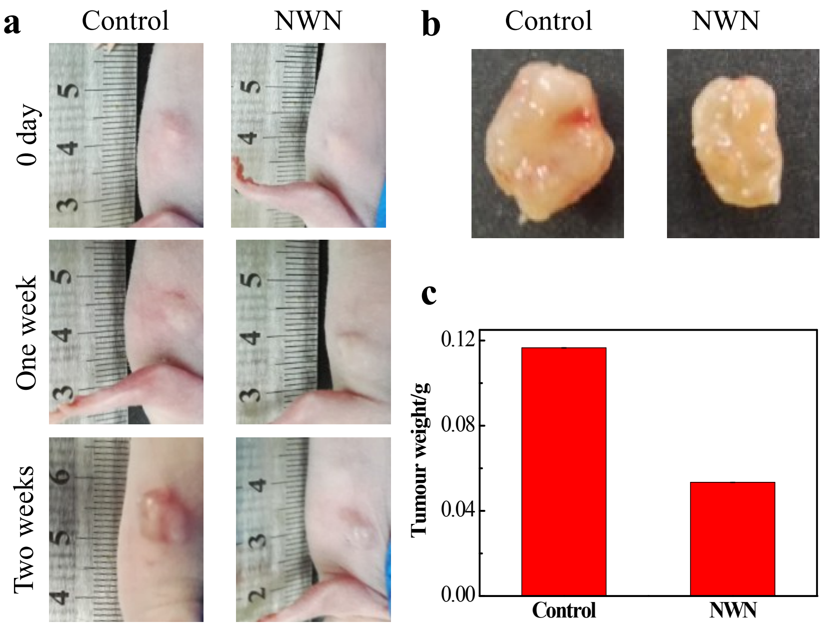

Figure S10. *In vivo* inhibition of tumor growth with NWN. (a) Photographs of tumors with different treatments at day 0, 7 and 14. (b) Photographs of tumors separated from nude mice with different treatment. (c) Average weight of the tumors.
